# 314. Six-Month Post-Acute Sequelae of COVID-19: High Self-Reported Morbidity among Women and Younger Adults

**DOI:** 10.1093/ofid/ofab466.516

**Published:** 2021-12-04

**Authors:** Ashish Bhargava, Wei Zhao, Mamta Sharma, Susan M Szpunar, Louis Saravolatz

**Affiliations:** 1 Ascension St John, Grosse Pointe Woods, MI; 2 Ascension St John Hospital, Gross Pointe Woods, Michigan; 3 Ascension | St John Hospital & Medical Center, Grosse Pointe Woods, MI; 4 Ascension St. John Hospital, MI; 5 St John Hospital, Detroit, Michigan

## Abstract

**Background:**

Long term sequelae across multiple medical domains, including the respiratory, psychiatric, and neurocognitive have been reported after COVID-19. Studies evaluating the impact of this symptom burden, however, are lacking. We aimed to describe the self-reported occurrence of symptoms and their effect on patient functioning six months after their acute hospitalization for COVID-19.

**Methods:**

From a historical cohort study of patients hospitalized for COVID-19 between March 8, and June 14, 2020, we identified patients discharged home. The purpose of the study was explained, and they were asked to consent to a telephone questionnaire. We used a modified version of a previously validated general symptom questionnaire (GSQ-30) to assess multi-system symptom burden. The Patient Health Questionnaire-2 (PHQ-2) was used to screen for major depression.

**Results:**

Of the original 565 patients, 258 patients were discharged home (45%). Of these, 57 (22%) patients were able to be contacted and agreed to participate in the survey. The mean (SD) age of the respondents was 55.1 (14.8) years, and 37 (64.9%) were female. The most common symptoms at follow-up were fatigue (60.0%), dyspnea (57.1%), feeling irritable, sad or decreased pleasure (56.4%), and memory difficulty (56.4%). Females had a significantly higher GSQ score (0.02) than males. Patients ages < 60 years tended to experience similar, if not greater, impaired functioning (p=0.07) compared with those ages ≥ 60 years (Table 1). Females were more likely to be irritable or sad (p=0.007), not feel rested on awakening (p=0.04), have shooting, stabbing and burning pain (p=0.02), have discomfort with normal light and sound (p=0.04), and have memory difficulty (p=0.04) than males (Table 2).

Table 1. Self-Reported Post-Acute Sequelae of COVID syndrome in adults younger than 60 versus adults at or older than 60 Years. SD: Standard deviation, ICU: Intensive care unit, ED: Emergency department, GSQ - General symptom questionnaire, PHQ-2: Patient Health Questionnaire-2

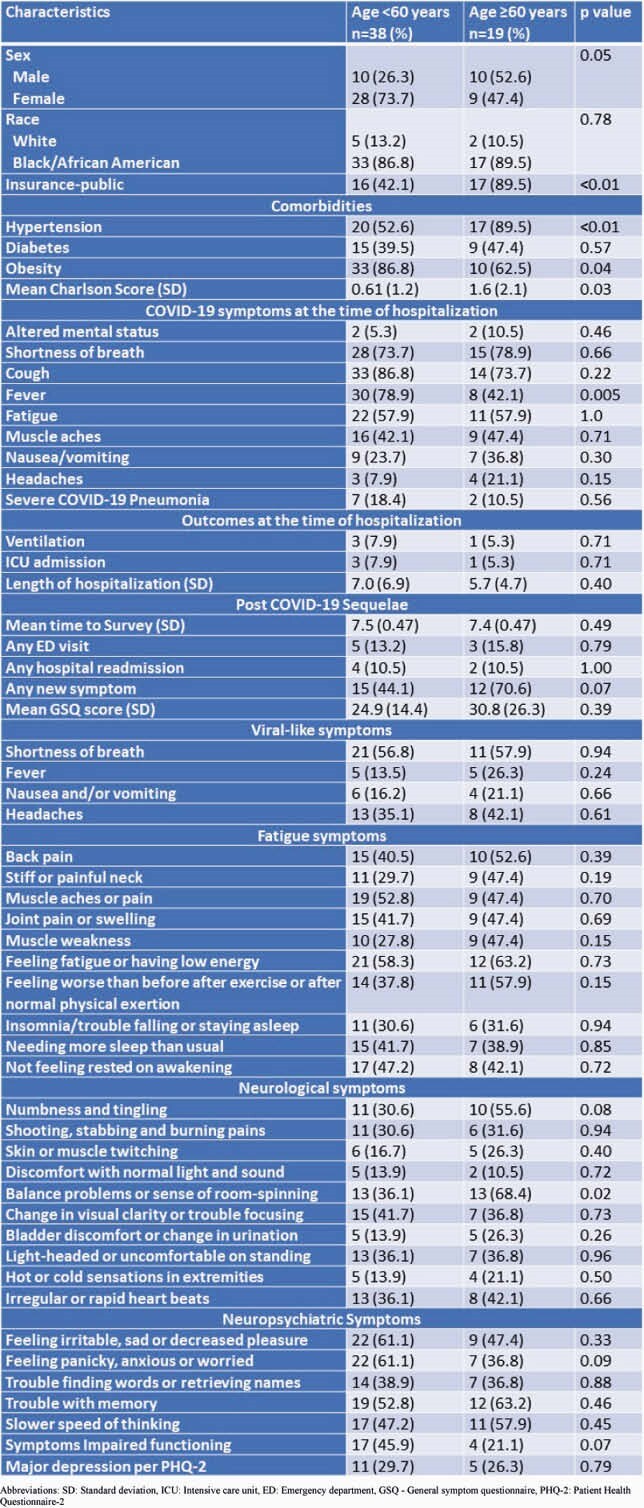

Table 2. Self-Reported Post-acute Sequelae of COVID syndrome in female versus male adults. SD: Standard deviation, ED: Emergency department, GSQ - General symptom questionnaire, PHQ-2: Patient Health Questionnaire-2

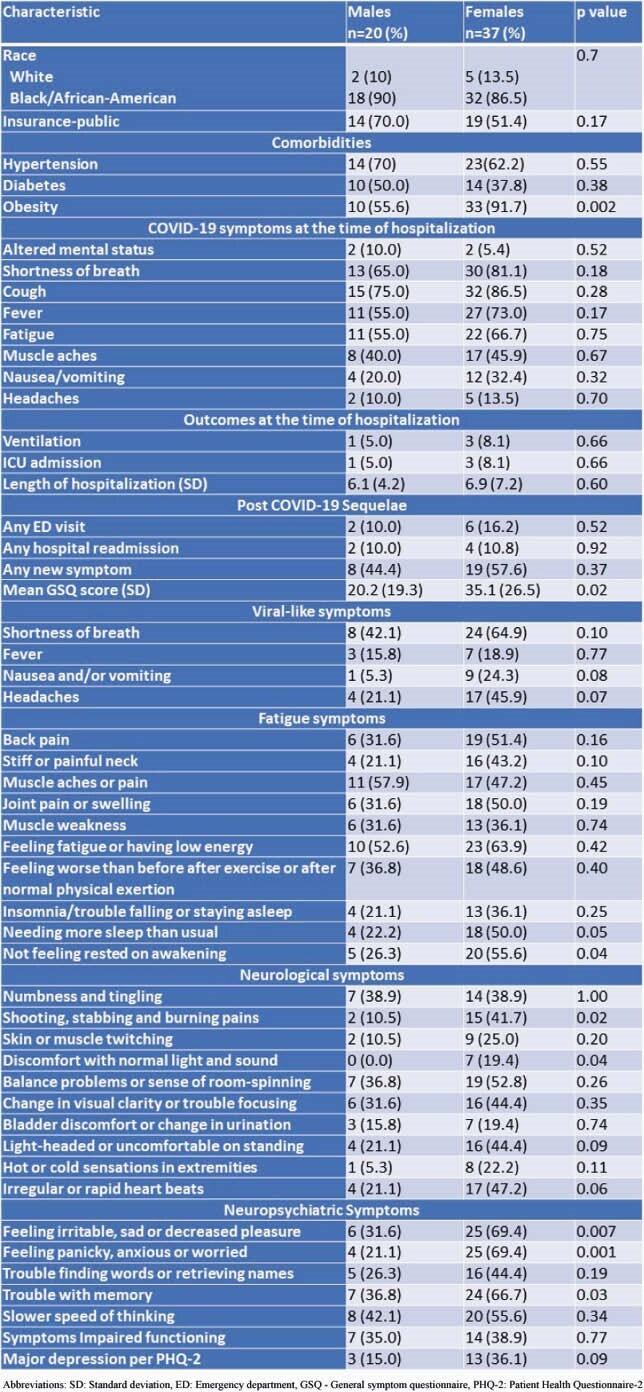

**Conclusion:**

Our study describes the clinical burden of post-acute sequelae of COVID-19 (PASC) in four core domains: fatigue, neurologic, neuro-psychiatric and viral-like symptoms. Over 45% of patients ages < 60 years suffered impaired functioning, compared with 21.1% of patient’s ages 60 years and above. Females had significantly higher GSQ scores than men which strongly corelates with the functional impairment among the females. Larger studies are needed to further validate our findings.

**Disclosures:**

**All Authors**: No reported disclosures

